# Using unstable data from mobile phone applications to examine recent trajectories of retail centre recovery

**DOI:** 10.1007/s44212-022-00022-0

**Published:** 2022-12-20

**Authors:** Patrick Ballantyne, Alex Singleton, Les Dolega

**Affiliations:** Department of Geography and Planning, University of Liverpool, Liverpool, England

**Keywords:** Retail Centre, Recovery, Mobility data, COVID-19

## Abstract

The COVID-19 pandemic has changed the ways in which we shop, with significant impacts on retail and consumption spaces. Yet, empirical evidence of these impacts, specifically at the national level, or focusing on latter periods of the pandemic remain notably absent. Using a large spatio-temporal mobility dataset, which exhibits significant temporal instability, we explore the recovery of retail centres from summer 2021 to 2022, considering in particular how these responses are determined by the *functional* and *structural* characteristics of retail centres and their regional geography. Our findings provide important empirical evidence of the multidimensionality of retail centre recovery, highlighting in particular the importance of composition, e-resilience and catchment deprivation in determining such trajectories, and identifying key retail centre *functions* and regions that appear to be recovering faster than others*.* In addition, we present a use case for mobility data that exhibits temporal stability, highlighting the benefits of viewing mobility data as a series of snapshots rather than a complete time series. It is our view that such data, when controlling for temporal stability, can provide a useful way to monitor the economic performance of retail centres over time, providing evidence that can inform policy decisions, and support interventions to both acute and longer-term issues in the retail sector.

## Introduction

The COVID-19 pandemic has caused significant damage across societies and economies around the world (Duong et al., 2022). As a result of policy actions imposed at various stages to mitigate the spread of the disease, the pandemic has severely disrupted daily activities, and has, and continues to change those ways in which we shop (Sit et al., 2022). This has had notable consequences for physical spaces of consumption such as high streets and retail centres, which have struggled for many years prior to the pandemic (Dolega & Lord, 2020). Within the UK, and in advance of COVID-19, vacancy rates were at an all-time high since the 2008 economic crisis (Wrigley et al., 2015), and footfall was significantly down (HSTF, 2021), in part due to the increasing popularity of online shopping and out-of-town shopping centres (Enoch et al., 2022). However, there is now a growing evidence base that the pandemic has accelerated these trends, often being likened to a ‘pandemic retail apocalypse’ or ‘catalyst for change’ (Frago, 2021).

Despite a wealth of literature exploring the short and medium-term impacts of public health restrictions on the retail sector (Baker et al., 2020; Nicola et al., 2020; Bonaccorsi et al., 2020), there has thus far been limited efforts to directly quantify these responses for retail centres, accounting for spatial heterogeneities at the regional level, and their *functional* and *structural* characteristics. The focus of this paper is therefore on British retail centres – “the primary sites of consumption in urban areas” (Dolega & Celinska-Janowicz, 2015, p.9), and their recovery from the initial shock of the COVID-19 pandemic. Although some examples for retail centres have emerged in cities (Frago, 2021; Ballantyne et al., 2022), these studies have emphasised the consequences of public health restrictions on retail centre activity, with much less written about the more recent ‘phases’, such as the Omicron variant. The latter is of great interest, as the Omicron subvariant re-infected many of those who were already vaccinated or had previously tested positive (Chowdhury et al., 2022; Grabowski et al., 2022), but saw no further public health restrictions, only recommendations.

In addition, existing studies have utilised various forms of data to assess the economic performance of consumption spaces, such as vacancies (Frago, 2021; Dolega & Lord, 2020) and footfall (Philp et al., 2022; Ntounis et al., 2020). The utility of mobility data in answering such questions was first identified in Trasberg and Cheshire (2021), providing significant scope for the use of similar data, such as the Geolytix aggregated in-app location dataset (CDRC, 2021a), to unpack how such responses have manifested in later phases of the COVID-19 pandemic once the key limitations of such data have been addressed. Thus, within this context, we explore the utility of a mobility dataset for exploring spatio-temporal trends of retail centre recovery, demonstrating in particular *how* we can use retail centre definitions and new forms of data as geographic data tools, to better understand the response of the wider retail sector to the pandemic. As such, we propose three research aims:

i) Consider the utility of the Geolytix mobility dataset for spatio-temporal analysis of retail centre recovery.
ii) Explore the extent to which these recovery trajectories relate to the overall *function*, and regional geography of retail centres.
iii) Quantify the role of the *structural* characteristics of retail centres, in addition to *function* and regional geography, in determining such recovery trajectories.

## Background

### The British retail (centre) landscape

The British retail landscape has undergone a large transformation. Driven in part by the rising popularity of e-commerce (ONS, 2022), the expansion of out-of-town developments and economic ‘shocks’ like the 2008 recession (Dolega & Lord, 2020), we have seen a significant decline of traditional high streets and retail centres (Wrigley et al., 2015). As a result, vacancy rates are at an all-time high, with increasing unemployment and concentration of retail away from high streets (Jones & Livingstone, 2018; Parker et al., 2017). The COVID-19 pandemic represents another challenge, significantly reducing footfall in many consumption spaces, following implementation of mobility restrictions to contain the spread of the virus (Enoch et al., 2022). Whilst these factors are well acknowledged as being some of the primary drivers of ‘brick-and-mortar’ retail decline, research suggests that these impacts are spatially heterogenous, with retail (centre) vulnerability and decline being highly variable, driven by multiple factors related to the *structur*al and *functional* attributes and catchment characteristics of the centres (Dolega & Lord, 2020; Singleton et al., 2016). What remains clear however is that the decline of retail centres is a multidimensional issue, which becomes increasingly convoluted when studied at different spatial scales, highlighting the complexity and diversity of the problem (Parker et al., 2017).

### Measuring retail centre performance

Although complex to capture, there is widespread consensus that data-driven empirical measures of performance hold great value for policy and planning of the future of cities and retail (Enoch et al., 2022; Philp et al., 2022). There are however no uniform indicators for measuring retail centre performance (Dolega & Lord, 2020), owing to the complexity of such a measure, and the influences of internal and external factors (Philp et al., 2022), as well as demand and supply (Jones et al., 2022). Total spend would be of greatest utility, but is difficult to obtain or estimate given the decentralised nature of retail (er) organisation. As such, there are numerous proxy measures that have been used, such as vacancy rates (Dolega & Lord, 2020; Jones et al., 2022), footfall (Philp et al., 2022; Ntounis et al., 2020) or attractiveness and retail mix (Dolega et al., 2016; Jones et al., 2022). However, such measures are subject to limitations, such as overly privileging certain geographic areas or having limited temporal resolution.

The increasing availability of new forms of data, creates novel opportunities for the monitoring of human mobility (Calafiore et al., 2022), and derivation of proxy performance measures for different places and spaces (Ballantyne et al., 2021), through which to understand urban problems. A large body of research is emerging that uses mobility data obtained from mobile phone applications to investigate human behaviour during the COVID-19 pandemic, notably changes in human mobility and internal migration (Kang et al., 2020), and the compliance of social distancing measures (Oliver et al., 2020). In addition, such data has been used to monitor the performance of consumption spaces and the wider retail sector during the pandemic (Trasberg & Cheshire, 2021; Ballantyne et al., 2021; Ballantyne et al., 2022).

However, mobility data is not without limitations. Location data from smartphones face similar challenges to other consumer datasets in that they are often unrepresentative of particular social groups (e.g. generational biases), or of particular areas due to differences in access to mobile devices/internet (Trasberg & Cheshire, 2021; Parsons, 2020). In addition, such data often faces significant temporal limitations, depending on the sample of devices and applications used to collect it, and their representativeness of the general population (Gibbs et al., 2021). Typically, the panel of unique devices will vary over time, which must be accounted for when using such data to conduct any spatio-temporal analysis (Trasberg & Cheshire, 2021). Thus, such mobility data is subject to its own limitations, and uncertainty in the generalisation of any results generated remains a significant challenge (Shi et al., 2022; Gibbs et al., 2021). However, there is still more to be unpacked about how such datasets can be used to monitor the economic performance of retail centres, particularly their post-pandemic recovery trajectories, and how these link to their overall *functiona*l, regional and *structural* characteristics.

### Retail centre performance and recovery

Observations about the short-term responses of retail centres to the pandemic, and the different restrictions and rules, form an essential basis to informing the preparedness of these locales in the future (Enoch et al., 2022). Much literature has focused on the consequences of restrictions during the earliest stages of the pandemic here in the UK. For example, Ntounis et al. (2020) and HSTF (2021) documented significant decreases in national footfall, whilst others identified notable disparities between different retailers (Baker et al., 2020; Nicola et al., 2020). Recently however, studies have emerged that examined these trends between different spaces of consumption, such as Enoch et al. (2022), who identified significant differences in footfall declines between UK town centres. Of great interest is how these disparities of impact and recovery relate to the characteristics of the retail centres, in particular their *functional* role (i.e., hierarchical positioning) and *structural* characteristics (e.g., vacancy rates). With *function,* studies have identified significant differences in responses between smaller, local centres and larger towns and cities (HSTF, 2021; Enoch et al., 2022; Ballantyne et al., 2022; Frago, 2021), relating these trends to the role of commuting, goods or scale of demand in determining such responses. With *structure*, research has identified significantly different responses depending on the vacancy rate, resilience to online shopping (e-resilience hereafter), diversity of retail offer and catchment deprivation of different retail centres (Enoch et al., 2022; HSTF, 2021; Dolega & Lord, 2020). Furthermore, related research has argued that such responses will exhibit significant spatial heterogeneities (Dolega & Lord, 2020), thus the importance of geographical location (e.g., regional geography) cannot be overlooked.

However, all of the above examples have examined the responses of retail centres to the earliest ‘phases’ of the COVID-19 pandemic, with much less written about more recent ‘phases’, such as that seen over the past year, where the Omicron subvariant has been of great significance. In the UK, the pandemic has been characterised by different sets of restrictions during different time periods, in response to different variants of the original virus. However, following Omicron, the government unveiled a much less stringent set of restrictions – “Plan B”, comprising mandatory face masks and vaccine passports (Prime Minister’s Office, 2021), with these being lifted in January. Thus, the likely supply side impacts on consumption spaces were greatly curtailed in comparison to restrictions seen earlier in the pandemic, theoretically enabling recovery to begin at the start of 2022, though this may have differed between different countries (e.g., Scotland, Wales). Thus, the overarching objective of this paper is to examine how retail centres responded beyond national lockdowns, how these relationships map into recovery (or decline) trajectories across different *functional*, regional and *structural* characteristics, and the utility of mobility data for capturing such trends.

## Data and analysis

### Geolytix ‘aggregated in-app location dataset’

The primary dataset used in this research; Geolytix *‘aggregated in-app location dataset’*, was obtained from the Consumer Data Research Centre (CDRC, 2021a). The dataset contains aggregated activity counts derived from in-app mobile phone applications across Great Britain, which are aggregated into a hexagonal geometry (H3), providing a count of the total number of distinct devices within each 50 m hexagonal cell. The data provides hourly, daily and weekly counts, spanning a 365-day period from August 2021 to July 2022, with the best spatial coverage occurring in towns, cities and other urbanised areas. It is important to note however that we are unable to identify the specific sources of data used to construct it (i.e., apps), as that information is commercially sensitive (CDRC, 2021a). For the purposes of our research in examining the response of retail centre activity, and to minimise disclosure risk, the mobility dataset was appended to the latest iteration of the CDRC retail centre boundaries (Macdonald et al., 2022); the nested H3 cells within each centre boundary were derived and joined with the corresponding mobility data, keeping only data within the centre boundary, before calculating the total number of devices within each retail centre at the weekly scale, as a proxy measure for retail centre activity, to smooth variation at the daily level.

**Fig. 1:**
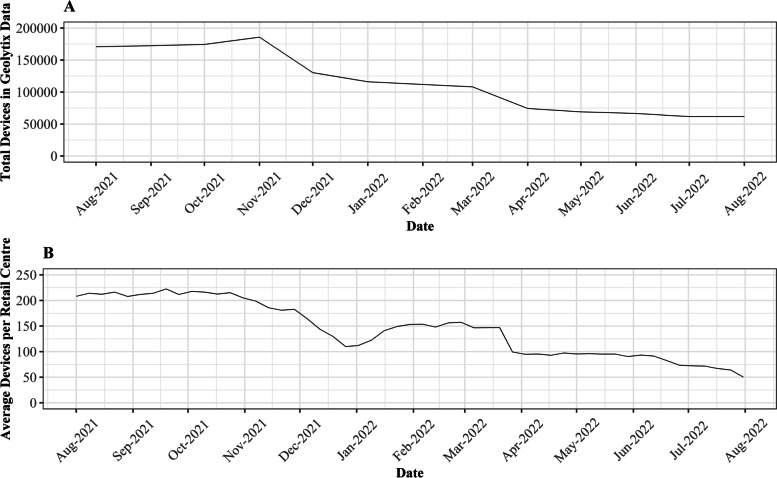
Changes in the number of devices in the Geolytix aggregated in-app location dataset throughout the study period, where 1A) demonstrates the falling number of devices in the sample and 1B) highlights its implications on the average number of devices within retail centres at the weekly scale

However, the temporal stability of the Geolytix mobility data remains a significant limitation, as is often the case with other similar mobility datasets (Trasberg & Cheshire, 2021). As demonstrated below in Fig. 1 A, the number of unique devices in the Geolytix mobility dataset does not remain consistent throughout the entire study period, falling from around 170,000 in August 2021 to 65,000 by July 2022, for reasons which are unavailable to us as users rather than data providers or creators. Thus, it is no surprise that a decreasing number of devices in the sample over time results in decreasing average device numbers within retail centres, as below in Fig. 1 B. This raises significant questions about the suitability of the Geolytix mobility dataset for analysis of trends over time, as any temporal trends are likely to be heavily affected by the decreasing number of devices in the sample. However, upon consultation with Geolytix, it was suggested that this decrease of devices does not compromise the representativeness of different geographical areas (i.e., regions) and types of retail centre (i.e., *functions*), when examining temporal trends at short time-periods such as weeks. Evidence of this can be seen below in Section 4.1 where we undertake a representativeness and stability analysis of the Geolytix data, before concluding that its stability between different regions and *functions* enables us to make robust comparisons between retail centres in Sections 4.2 and 4.3.

### Supporting datasets

To investigate the role of retail centre *function* and *structure* in determining the response of retail centre activity during the study period, we utilised the safeguarded CDRC ‘retail centre indicators’ data product (CDRC, 2021b), which provides summary indicators for the retail centres. Specifically, to characterise the *function* of the centres, we utilised the retail centre hierarchy (Classification), as described below in Table 1. To characterise the *structure* of the centres, we utilised the remaining indicators, listed below in Table 2, comprising information about the composition, diversity, catchment deprivation and e-resilience of the retail centres. As the retail centre indicators are available only for a subset of the 6423 retail centres in Great Britain, only those retail centres for which both *functional* and *structural* indicators were available were used in this investigation. This results in exclusion of the large number of small centres across Great Britain and some additional larger centres (retail parks, shopping centres), for which indicators are not available and/or are significantly different in *function* and *structure* (CDRC, 2021b; Jones et al., 2022). These exclusions were not desirable; however, this step was unavoidable as disclosure risk means these indicators are not available for these very small retail centres. The result was a set of 1068 study retail centres across the UK, comprising weekly data on retail centre activity (i.e., total devices_)_ and the accompanying *functional* and *structural* indicators for the retail centres.

**Table 1:** Retail centre hierarchy (Classification), describing the *functional* differences between the retail centres, obtained from CDRC (2021b)

Classification	Examples	N
Regional Centre	London, Birmingham City, Liverpool City, Manchester City, Glasgow City.	14
Major Town Centre	Carlisle, Warrington, Luton, Bournemouth, Swansea.	82
Town Centre	Grimsby, Welwyn Garden City, Clapham Junction, Torquay, Tenby.	270
District Centre	Ellesmere Port, Camden Town, Chesham, Greenside.	228
Market Town	Berkhamstead, West Kirby, Bakewell, Kenilworth, Billericay.	112
Local Centre	Newport Pagnell, Frodsham, Oadby, Egham.	378

**Table 2:** Retail centre *structural* indicators, obtained from CDRC (2021b)

Variables	Description	
propChain	Proportion of chain retailers	
propIndependent	Proportion of independent retailers	
pctCloneTown	Proportion of ‘clone’ retailers	
propVacant	Proportion of vacant retailers	
propStructuralVacant	Proportion of vacant retailers since 2017	
propVacantChange	Change in vacancy from 2017 to 2020	
propComparison	Proportion of comparison retailers	
propConvenience	Proportion of convenience retailers	
propService	Proportion of service retailers	
propLeisure	Proportion of leisure retailers	
onlineExposure	Online exposure score (Singleton et al., 2016)	
vulnerabilityIndex	Vulnerability index (Singleton et al., 2016)	
eResilience	Composite e-resilience index (Singleton et al., 2016)	
AvgIMDScore	Average IMD score of walking catchment	
IMDDecile	Corresponding (national) decile for average IMD score	

### Analytical approach

**Fig. 2:**
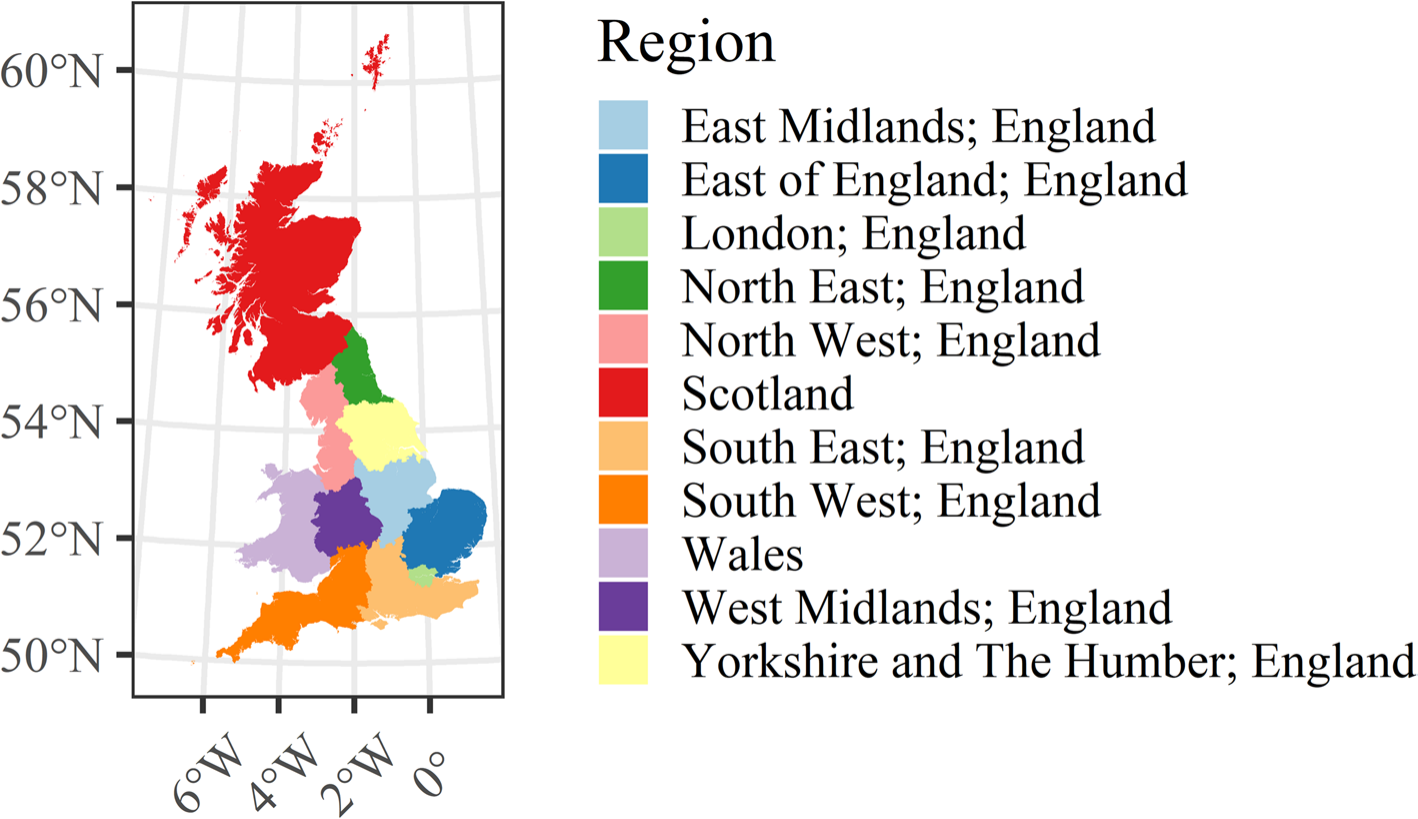
UK Regions, excluding Northern Ireland for the purposes of this study, as no retail centre indicators are available for retail centres in Northern Ireland

The economic performance of consumption spaces is a product of numerous forces of change, making it a highly complex problem to understand (Parker et al., 2017), and as we have highlighted thus far, existing research shows that the *functional* role, *structural* composition and regional geography are all linked to the overall performance of retail centres both in the short and longer term. Thus, in Section 4.2, following formal validation that the number of devices remained stable at the weekly scale and between different regions and *functions* as suggested by the data provider (Section 4.1), we explore how retail centres with differing *functions* and in different regions (see Fig. 2) have responded during this study period, examining changes to activity within them. In particular, once we have controlled for specific regional biases created by the mobility data, we examine changes to retail centre activity as share change between different *functions* and regions, as it would not be appropriate to visualise change in total or average devices over time, as these trends would be subject to underlying limitations of the data (Section 3.1).

Finally, in Section 4.3 we seek to unpack the role of the *structural* characteristics of retail centres in determining their response during this time, through implementation of a modelling framework to quantify the impacts of different *structural* and catchment characteristics on changes to activity during this time, as well as considering how retail centre type (*function*) and region are related to such trends. In particular, we model the relationship between these independent variables and the change in share of total devices (i.e., activity) from a baseline (August–September average) to summer 2022 (June–July average). Thus, for every retail centre we have the change in activity from 2021 to 2022 (∆_i_) as the dependent variable, and the *functional,* regional and *structural* attributes of the retail centre as the independent variables, as outlined in Eqs. 1 and 2 below.

1 $${\Delta }_i={\beta}_0+{\beta}_1\dots {\beta}_9+\varepsilon$$

Eq 1 Model specification for *structural* (and catchment) characteristics of retail centres, following collinearity assessment of all variables in Table 2 (see Section 4.3)

2 $${\Delta }_i={\beta}_0+{\beta}_1\dots {\beta}_9+{\beta}_{10}+{\beta}_{11}+\varepsilon$$

Eq. 2 Model specification for *structural, functional* and regional characteristics of retail centres. Reference categories for β_10_ and β_11_ were Local Centres and Yorkshire and The Humber, due to low variation below in Section 4.2

Where:

∆_i_ = change in share of total devices between all retail centres nationally (%) from Aug/Sept 2021 to June/July 2022 for retail centre *i* (*continuous*).

β_1_ = pctCloneTown (*continuous*, see Table 2).

β_2_ = propVacant (*continuous*, see Table 2).

β_3_ = propVacantChange (*continuous*, see Table 2).

β_4_ = propComparison (*continuous*, see Table 2).

β_5_ = propConvenience (*continuous*, see Table 2).

β_6_ = propLeisure (*continuous*, see Table 2).

β_7_ = propService (*continuous*, see Table 2).

β_8_ = eResilience (*continuous*, see Table 2).

β_9_ = AvgIMDScore (*continuous*, see Table 2).

β_10_ = *function* of retail centre *i* (*ordinal*, see Table 1).

β_11_ = *region* that retail centre *i* is located in (*nominal*, see Fig. 2).

## Findings

### The utility of Geolytix mobility data

**Fig. 3:**
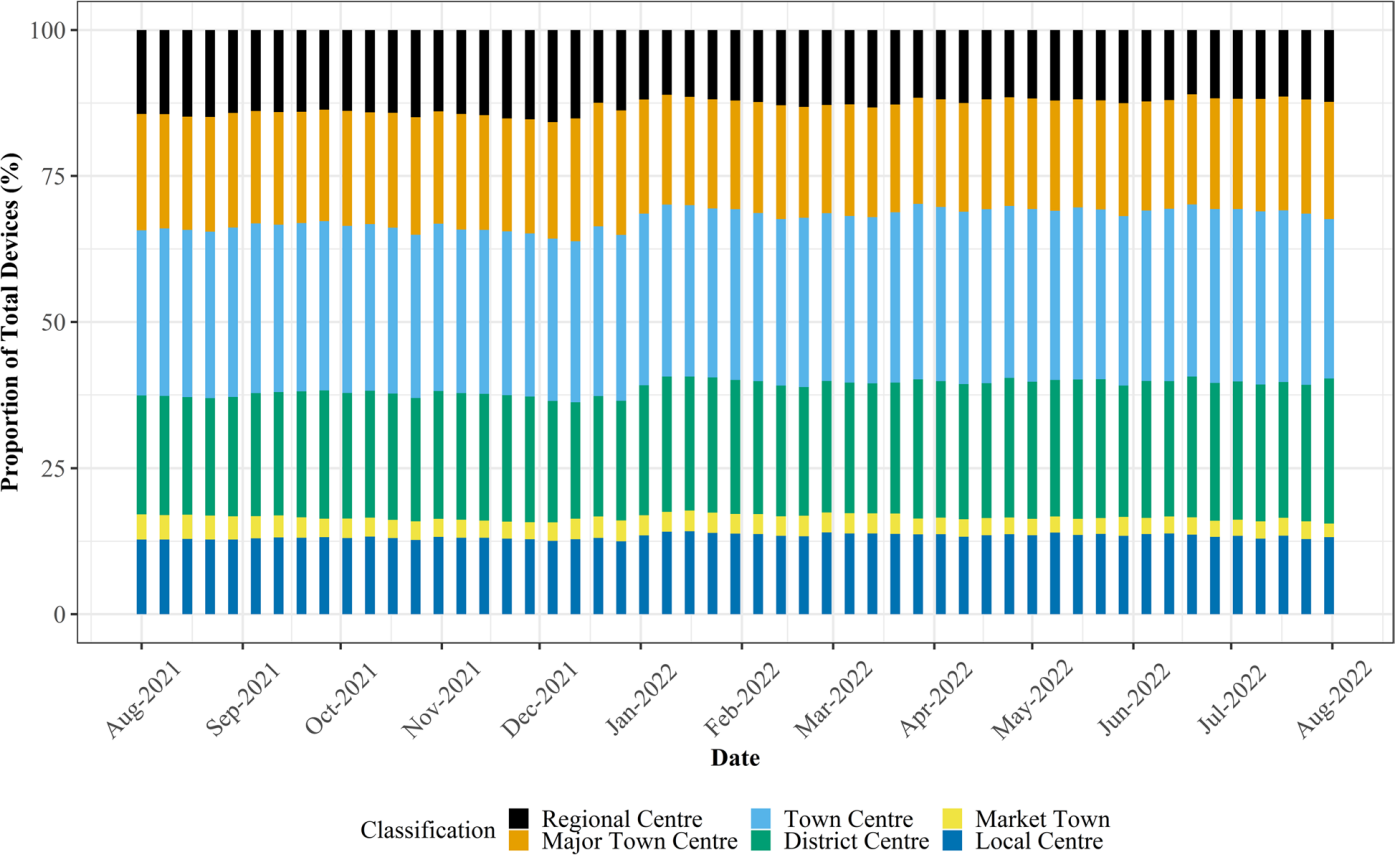
Stability of devices between different retail centre functions, highlighting the consistent share of devices between the six retail centre types over the study period

**Fig. 4:**
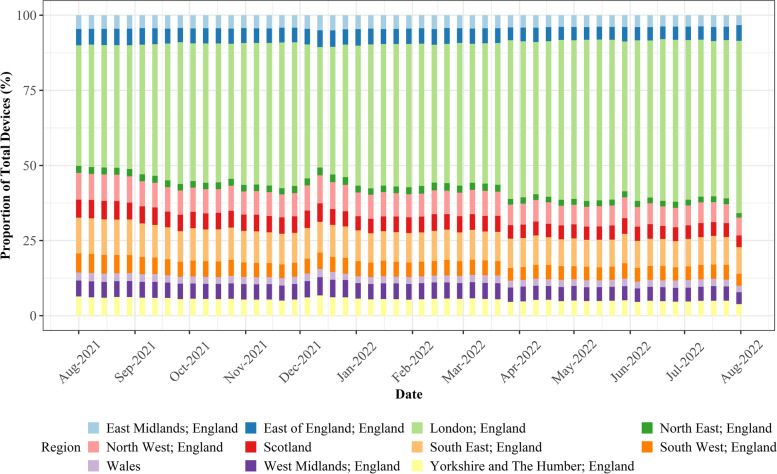
Stability of devices between different UK regions, highlighting the increasing share of devices in retail centres located in London over the study period, and general stability of device decline in all other regions

As discussed in Section 3.1, significant attention must be paid to the representativeness and temporal stability of mobility data when seeking to explore temporal trends. Following direct consultation with Geolytix, it was suggested that their mobility data exhibits significant stability across days and weeks and between different regions, retail centre *functions* and directly comparable retail centres, despite a falling number of devices across the entire sample. To validate this and ensure our analysis did not fail to account for the changing number of devices, we calculated the proportion of national devices allocated to individual *functions* and regions at the weekly level to smooth variation in daily trends (Figs. 3 and 4), helping us to identify whether robust comparisons could be made between retail centres, despite changing devices in the sample.

From Fig. 3 it is apparent that in terms of retail centre type (i.e., *function*), whilst the total number of devices in the sample fell dramatically over the study period (Fig. 1 A), the proportion of devices in each of the six types of retail centre remained largely consistent over the study period. This highlights that the mobility data does not bias certain types of centres, providing justification for comparison of share change in activity between different *functions* over time, as below in Section 4.2. In contrast, Fig. 4 clearly illustrates that the loss of devices over the study period had a very distinct geography; it appeared to create a significant bias in London, where centres occupied a greater share of total devices nationally. However, we cannot be certain why this is occurring, as it could relate to movement of people back into London following the pandemic, or the growing popularity or accessibility to certain mobile phone providers in London which are unknown to us, so we must control for this in some way. Thus, we will focus on recovery trajectories for all retail centres outside of London, as we can be confident that these trends are not subject to the inherent biases created by changing numbers of devices, resulting in a final sample of 862 retail centres. Whilst retail centres in London might comprise a more stable sample, the other 10 regions experienced a consistent decline in the number of devices, so retail centres within these regions are directly comparable to each other, providing new insights, as opposed to existing literature on the response of retail centres to COVID-19 in London (Trasberg & Cheshire, 2021). However, what we are unable to do is explore individual retail centre trends over time, as they will be affected by the changing number of devices in the sample. Instead, we can compare retail centres within certain *functions* or in certain regions, as they have not been directly biased by this change in underlying devices, once London has been controlled for.

### Exploring the response of retail centres

**Fig. 5:**
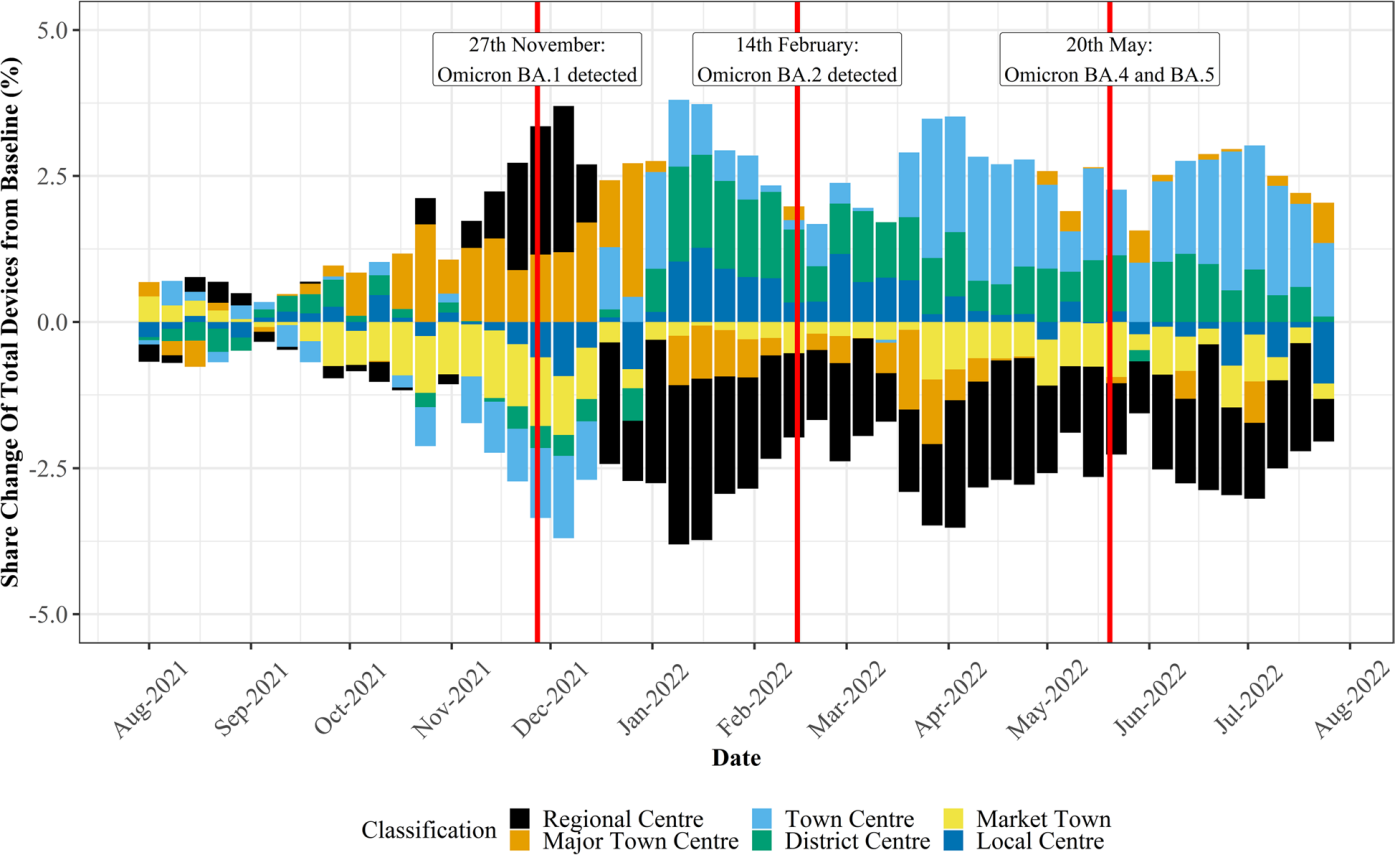
The functional response of retail centres visualised as the change in share of total devices (%) from the baseline, defined as the average share of devices (%) by retail centre type in August and September 2021

The response of different types of retail centres across the study period, as seen below in Fig. 5, was of great interest. Firstly, there appeared to be no direct response to the arrival of Omicron in late November 2021 or its subvariants in February and May 2022, with the overall share of total devices between the six types of retail centre remaining largely unchanged in response to those key dates. This suggests that Omicron did very little to abruptly change the types of places people chose to shop, a direct contrast to what has been seen in earlier phases of the COVID-19 pandemic (Harris, 2022; Enoch et al., 2022; Ballantyne et al., 2022; Frago, 2021). However, across the entire study period, there were interesting shifts in the change of share between the retail centre types, which raise significant questions about the longer-term recovery of different retail *functions*.

For example, if we look specifically at Regional Centres, the largest in size and typically the most diverse in retail offer (Macdonald et al., 2022), they exhibited a significant increase in share in the lead up to Christmas 2021, an expected trend given that these centres comprise the largest variety of retailers, products and ancillary activities, better fulfilling the needs of Christmas shoppers. However, what is most interesting is that following Christmas 2021, Regional Centres exhibited the most notable decline in share of activity from the baseline, suggesting that this specific *function* of retail centre has become less popular over the last year, relative to other retail centre *functions,* mirroring much of the literature seen earlier in the pandemic (Ballantyne et al., 2022; Frago, 2021). Whilst this trend could be a result of shifts in consumer behaviour in response to Omicron or the recent cost-of-living crisis, we can be sure that this trend is robust and not a product of falling devices in the Geolytix sample, given our examination of the stability of the dataset between different retail centre *functions* in Section 4.1. On the other hand, Town Centres saw a reversal of share following Christmas 2021, where their importance became more significant following the Christmas period, similar to District Centres and Local Centres. These trends are interesting as during the first half of 2022, the UK was under “Plan B” restrictions, which were implemented to control the spread of the virus. Whilst we cannot be certain, it’s not implausible to suggest that increasing activity in smaller retail centres (e.g., Local Centres) following Christmas and during 2022 was a result of risk-mitigation behaviours aiming to reduce exposure to Omicron during this time, as formal restrictions on mobility were not in place under “Plan B”. This links to literature from earlier phases of the pandemic, where those *functions* deemed to be lower risk through a more ‘localised’ *function*, were those to experience the least significant impacts during the early stages of COVID-19 (Enoch et al., 2022; Frago, 2021; HSTF, 2021).

**Fig. 6:**
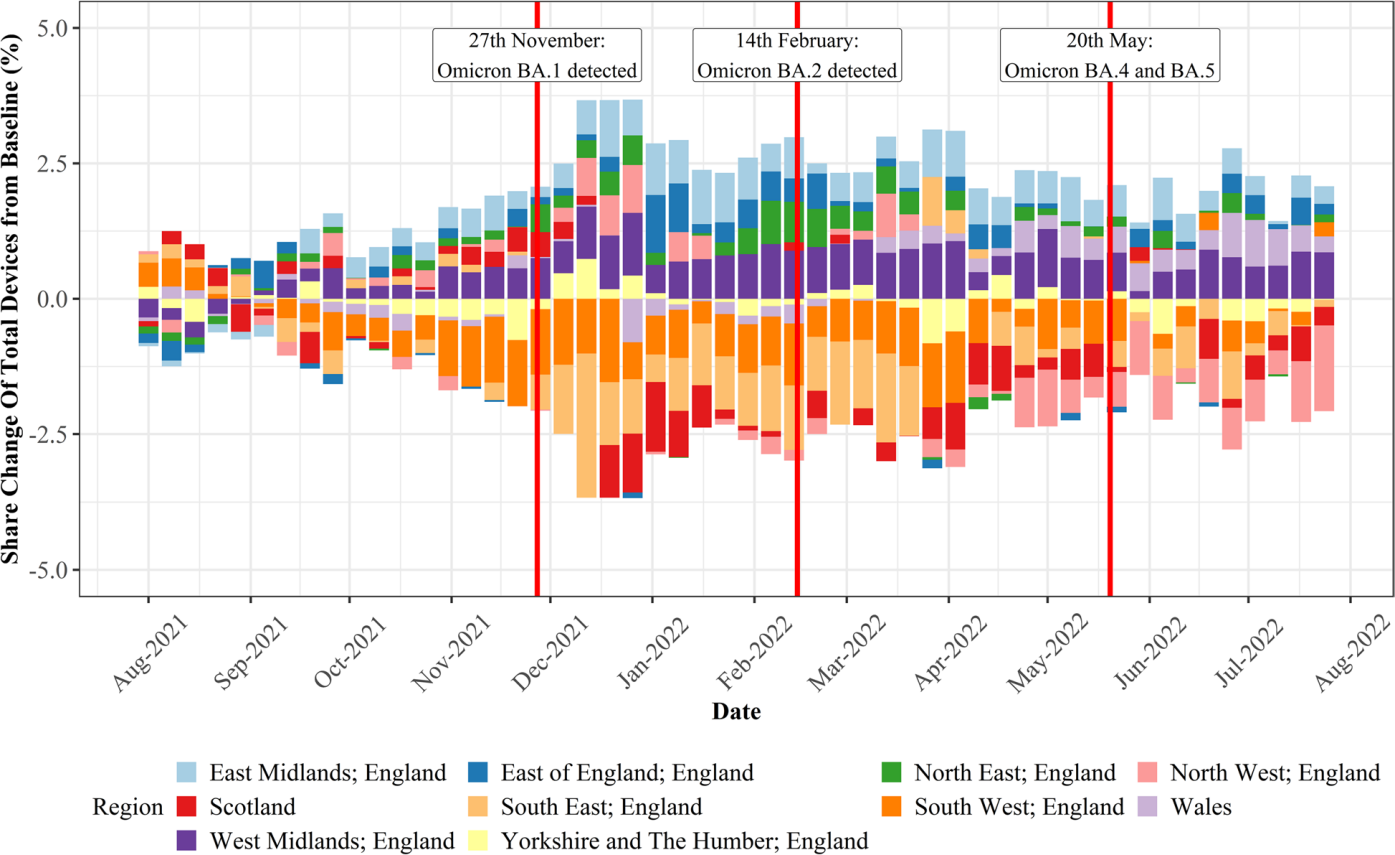
The regional response of retail centres visualised as the change in share of total devices (%) from the baseline, defined as the average share of devices (%) by region in August and September 2021

Similar trends can be seen when examining the recovery of retail centres in different regions too (Fig. 6), which was posited to be a strong determinant of the economic performance of retail centres (Dolega & Lord, 2020). The largest decreases in activity were seen for retail centres in the South, specifically the South East and West, with noticeable decreases also seen in the North West and in Scotland. On the other hand, retail centres in East Anglia, East of England and West Midlands all appeared to experience significant uplifts in activity, when compared against the baseline period. Thus, what remains clear from this section is that *functionally* and regionally, there are significant disparities in terms of the recovery of retail centres during this time, with significant inequalities in how these recovery trajectories are manifesting between retail centres. Such inequalities are however not fully understood following our exploratory analysis, as we have generalised the responses of retail centres based on *functional* and regional averages, instead of exploring individual responses.

**Fig. 7:**
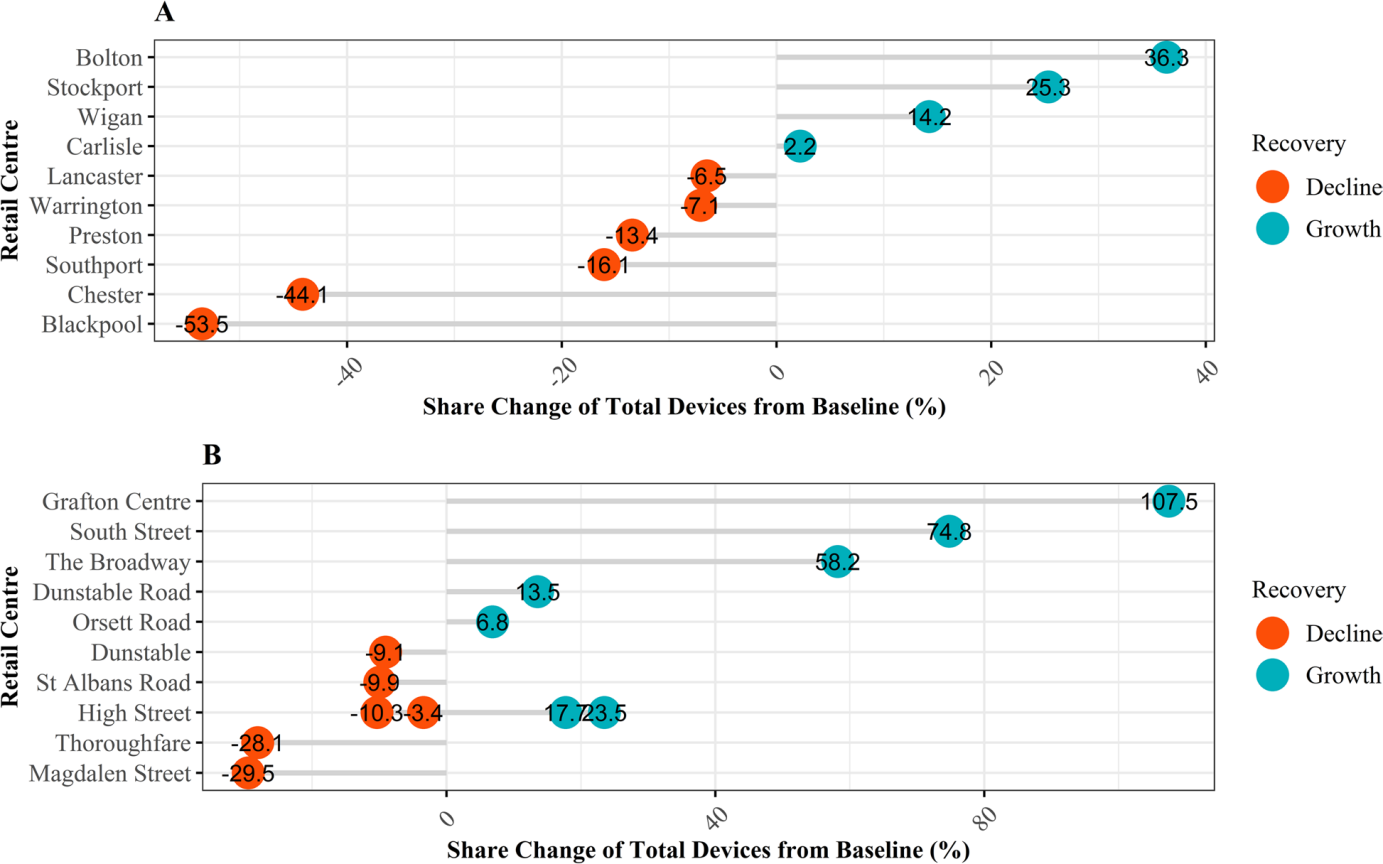
Recovery trajectories of Major Town Centres in the North-West (A) and District Centres in East of England (B). Trajectories have been calculated as the change in share of total devices (%) from the baseline, defined as the average share of devices (%) by region in August and September 2021

Thus, to demonstrate the importance of considering these trajectories at greater resolution, below we examine the individual responses of all Major Town Centres in the North-West (Fig. 7 A) and District Centres in the East of England (7B), highlighting the heterogeneity of responses between retail centres with the same *function* and regional geography. As above in Fig. 5, Major Town Centres at the national level appeared to be experiencing an overall period of decline as opposed to recovery when compared against other types of retail centre in the UK, which theoretically should be more dramatic for those in the North-West of England (Fig. 6). However, what is apparent from Fig. 7 is that there is significant variation between retail centres, and whilst the majority did experience decline over the study period, though to varying degrees, there were some retail centres that experienced growth. Similarly, when looking at District Centres in East Anglia, whilst the majority are experiencing growth, though to varying degrees, there are still numerous retail centres experiencing decline, contrary to the national-level trends identified in Figs. 5 and 6. Thus, this highlights the complexity of retail centre performance and recovery (Parker et al., 2017), which can be generalised to the national-level to provide a general overview of the role of *functional* and regional characteristics. However, significant variations in recovery clearly exist between individual retail centres that share similar characteristics, requiring analysis at a higher resolution to unpack some of these ideas. In addition, whilst *function* and region clearly interact with these trajectories, it is likely that the intrinsic *structural* composition of retail centres and their relationship to the catchment have a role too, as discussed in Section 2.3. Thus, an approach that can quantify these interactions more effectively is required, specifically one that can identify the relationships between *function,* region and *structure* on the trajectories of retail centre recovery, and quantify the importance of each.

### Modelling the response of retail centres

As above, further analysis is required to unpack the significant amount of variation seen between the recovery of individual retail centres. Thus, in this section we deploy a modelling framework, as described above in Section 3.3, to quantify the role of *function,* region and the *structural* characteristics of retail centres in determining their response over the study period. Firstly, we examined the prevalence of high collinearity between independent variables utilising correlation analysis. Highly collinear variables were identified based on two criteria; those which have been used to create another independent variable such as onlineExposure and vulnerabilityIndex which are put together to construct eResilience, or those where the correlation coefficient exceeded 0.7 or was lower than − 0.7. Following removal of *structural* characteristics with high collinearity, we fit a model (see Eq. 1) to first assess the role of the *structural* (and catchment) characteristics of retail centres in determining the change in activity between summer 2021 and summer 2022 (∆_i_), as described above in Section 3.3.

The results of a model fit with just the *structural* characteristics can be seen below in Table 3, where coefficients are interpreted as the estimated percentage change in retail centre activity (share of total devices) given a one-unit change in each of our explanatory variables. The results suggest that in general, the *structural* (and catchment) characteristics of retail centres are associated with ∆_i_, though to varying degrees. For instance, those with higher proportions of Leisure retailers were more likely to experience negative growth (− 0.735), as at the beginning of the COVID-19 pandemic (Enoch et al., 2022), whilst those with higher proportions of Service retailers were more likely to experience growth (0.830). This suggests that over the 12-month study period, retailers with a more ‘essential’ retail offering were those that occupied a greater share of consumers, which is supported by a positive coefficient for propConvenience and negative coefficient for propComparison, though both were not statistically significant. Whilst both statistically insignificant, the coefficients for variables describing the vacancy of retail centres were also of great interest; both exhibited negative coefficients suggesting that retail centres struggling with larger numbers of empty stores typically experienced negative growth during the study period, a well-documented determinant and consequence of the changing economic performance of retail centres (Dolega & Lord, 2020; Enoch et al., 2022).

Unsurprisingly, the resilience of retail centres to online shopping (eResilience) was seen to have a positive effect on the recovery of retail centres during this time; retail centres with a high resilience to online shopping came to occupy a greater share of consumers between 2021 and 2022. This is interesting, as in the UK the e-resilience of centres has long been considered a vital determinant of their economic performance both in and out of the COVID-19 pandemic (Singleton et al., 2016; Enoch et al., 2022), and appears to still be a key factor. This raises interesting debates about the continued plurality of different retail centres; those deemed to provide an offering that will not be overshadowed by online shopping (i.e., higher e-resilience), have recovered faster and appear to be maintaining such recovery, when compared with those more susceptible to the effects of e-commerce. This is a similar trend to what was seen in earlier phases of the pandemic, where large numbers of people were switching to online purchasing (Ntounis et al., 2020), only visiting stores/retail centres where they could access a good or service less suited to e-commerce, typically service and/or convenience retailers, both of which exhibited positive coefficients above in Table 3. From a conceptual standpoint, this is interesting as this measure accounts for the structural components and level of ‘supply’ (Singleton et al., 2016), but also incorporates catchment characteristics through quantification of the ‘online exposure’ of the catchment (i.e., demand), demonstrating the importance of understanding the role of supply and demand when trying to unpack the response of retail centres, and their economic performance, as in Jones et al. (2022).

**Table 3:** Model results for *structural* (and catchment) characteristics of retail centres

Variable	Coefficient	***p*** value	Sig.
pctCloneTown	0.151	0.390	–
propVacant	−0.304	0.523	–
propVacantChange	−0.158	0.764	–
propComparison	−0.433	0.167	–
propConvenience	0.467	0.448	–
propLeisure	−0.735	0.039	*
propService	0.830	0.018	*
eResilience	0.369	0.030	*
AvgIMDScore	0.429	0.011	*

Significance levels: < 0.05 *, < 0.01 **, < 0.001 ***, R-squared: 0.33, Adjusted R-squared: 0.23.

The final independent variable that exhibited a statistically significant association with change in activity was deprivation (AvgIMDScore), as initially suggested by Dolega and Lord (2020), where retail centres in more deprived areas were seen to occupy a greater share of consumers, i.e., recovering at a faster rate. This is an interesting finding, and the first to link the economic performance of retail centres directly to the deprivation of its catchment. A plausible explanation could relate to the implementation of “Plan B” recommendations, which occurred during the study period (November 2021 – February 2022) to reduce the spread of Omicron. It is well documented that neighbourhoods with differing socio-economic and demographic showed different levels of engagement with government restrictions and vaccination programmes throughout the pandemic (HM Government, 2022). This could be apparent here, where people in more deprived areas could have been less likely to follow to government recommendations and reduce their mobility during this time, resulting in higher activity in nearby retail centres, as above in Table 3.

Thus, we have identified some interesting associations between the *structural* characteristics of retail centres and their recovery trajectories over the study period. However, given our exploration of the response of retail centres with different *functions* and regional geography in Section 4.2, it is important that we incorporate such insights into the modelling framework to identify the concurrent role of *function,* region and *structure* in determining the response of retail centres. The results of the model with only the significant *structural* indicators from Table 3, and dummy variables for retail centre *function* and region can be seen below in Table 4 (see Eq. 2). The coefficients for region and *function* can be interpreted as the average change in retail centre activity for the comparison group relative to the reference group, keeping all other variables constant. The reference groups were selected as Local Centres (Classification) and Yorkshire and The Humber (Region), given their low variance across the study period, as identified in Section 4.2.

**Table 4:** Model results for the *structural, functional* and regional characteristics of the retail centres. Reference categories for Classification and Region are ‘Local Centres’ and ‘Yorkshire and The Humber’ respectively

Variable	Coefficient	p value	Sig.
propLeisure	−0.537	0.089	–
propService	0.968	0.001	*
eResilience	0.348	0.028	*
AvgIMDScore	0.164	0.050	*
*(Classification)* Regional Centre: Local Centre	−2.810	0.832	–
*(Classification)* Major Town Centre: Local Centre	−1.920	0.757	–
*(Classification)* Town Centre: Local Centre	2.620	0.510	–
*(Classification)* District Centre: Local Centre	4.580	0.320	–
*(Classification)* Market Town: Local Centre	−9.520	0.049	*
*(Region)* East Midlands: Yorkshire and The Humber	1.400	0.843	–
*(Region)* East of England: Yorkshire and The Humber	−6.960	0.303	–
*(Region)* North East: Yorkshire and The Humber	−1.270	0.886	–
*(Region)* North West: Yorkshire and The Humber	0.959	0.877	–
*(Region)* Scotland: Yorkshire and The Humber	−8.580	0.275	–
*(Region)* Wales: Yorkshire and The Humber	3.333	0.651	–
*(Region)* South West: Yorkshire and The Humber	−15.200	0.018	*
*(Region)* South East: Yorkshire and The Humber	−10.800	0.078	–
*(Region)* West Midlands: Yorkshire and The Humber	3.310	0.621	–

Significance levels: < 0.05 *, < 0.01 **, < 0.001 ***, R-squared 0.30, Adjusted R-squared: 0.25.

Similar to the earlier discussion, propService, eResilience and AvgIMDScore exhibited statistically significant positive associations with retail centre activity, which can be interpreted as increasing the overall recovery of retail centres during this time. In terms of the *function* of retail centres, the direction of the coefficients aligned with earlier findings about the recovery or growth of retail centres during this period; for example, retail centres classified as Major Town Centres, Regional Centres and Market Towns were all found to have negative associations with ∆_i_ on average, relative to Local Centres, as in Fig. 5. In comparison, District Centres and Town Centres exhibited positive associations, again matching the discussion in Section 4.2. However, it is important to consider these findings in relation to their statistical significance; very few retail centre *functions* exhibited statistically significant associations with the change in retail centre activity between 2021 and 2022; Market Towns were the only retail centres to exhibit a statistically significant relationship with ∆_i_. Whilst the use of share change (over total devices) could flatten the significance of *functional* differences in recovery, it appears that *functional* differences are of less significance than the *structural* and catchment characteristics of retail centres in determining recovery, an interesting finding.

Similarly, when looking at responses between regions (Table 4), the direction of the coefficients was again unsurprising, with those regions identified in decline earlier (Fig. 6) such as the South East, South West and Scotland all having negative coefficients, relative to Yorkshire and The Humber, though not all were statistically significant. What is particularly interesting is that the region that appeared to experience some of the most significant reductions in share in Fig. 6, the South West, had a statistically significant negative association with retail centre activity, detailing that retail centres in the South West were more likely to experience decline than recovery during this period, relative to the reference category and keeping all other indicators constant. However, as with *functional* responses, it is important to reiterate that most regions exhibited statistically insignificant relationships with ∆_i_ during the study period.

Thus, what remains clear from this modelling exercise is that retail centre recovery (∆_i_) during this time is dependent on the overall *structure, function* and regional geography of the retail centres, though to varying degrees, with *function* and regional geography contributing significantly less. It appears that the *structural* and catchment characteristics of retail centres remain a greater determinant of changes to retail centre activity during this time, thus more research is needed to unpack how at finer geographical resolutions (as opposed to regions), different *structural* characteristics of retail centres geographies determine such responses (Dolega & Lord, 2020; Philp et al., 2022). However, there are lots of additional unanswered questions that need addressing, such as the role of multidimensional typologies (e.g., Dolega et al., 2021), seasonal and weather effects (e.g., Rose & Dolega, 2022) and the recent cost-of-living crisis, which has exacerbated inequalities between different regions (Wood, 2019). Furthermore, it would be of great utility to identify how and when these recovery trajectories began, given data with a longer timescale, though this was not possible with the Geolytix data used in this investigation.

## Discussion and conclusions

Spaces of consumption such as retail centres have faced significant challenges in recent years, with the COVID-19 pandemic continuing to exacerbate the decline of physical retail spaces. Whilst some studies have explored the response of consumption spaces to the pandemic, they are often restricted to specific geographic areas, or tend to focus on the impacts of national lockdowns during the earlier waves of the pandemic. Using mobility data from Geolytix, we investigated the recovery of retail centres across Great Britain, during a period characterised by the Omicron variant. Our findings are of great significance, providing an overview of the response of retail centres at the national level for the first time, demonstrating that such responses were partially determined by the *functional, structural* and regional characteristics of the centres.

Perhaps the most important finding was that the response (and recovery) of retail centres was not homogenous, providing evidence that examination of national trends of retail centre recovery, as in Section 4.2, are not enough to capture variation in responses between a network of centres with different *functional,* regional and *structural* characteristics. By modelling the nature of these recovery trajectories between centres with different characteristics in Section 4.3, we highlight that there were specific ‘winners’ and ‘losers’ during the study period. Functionally, whilst retail centres towards the top of the hierarchy (e.g., Regional Centres) appeared to exhibit the most pronounced recovery leading up to Christmas 2021, this trends reversed in 2022, where the popularity of retail centres at the cores of major towns and cities saw decline rather than growth, as earlier in the pandemic (Ballantyne et al., 2022; Frago, 2021). In addition, we identified significant regional inequalities in retail centre recovery, such as the apparent decline of retail centres in the South (excluding London), whilst retail centres in the Midlands, Wales and areas of the North exhibited the opposite trend. Finally, we identified specific *structural* characteristics that were associated with stronger recovery; lower dominance of ‘non-essential’ retail (e.g., Leisure), higher resilience to online shopping and greater levels of deprivation within the catchment, with *structural* characteristics appearing to be a greater determinant of recovery than the overall *function* or regional geography of retail centres.

We must however remain cautious of these trends, especially given they are based on exploratory analysis and modelling, which did not account directly for the impacts of seasonality, weather and holiday periods (Lyu et al., 2022; Rose & Dolega, 2022), and is based on trends for a subset of the major retail centres across the UK. Further research should seek to identify what additional knowledge can be generated about retail centre recovery by focusing on retail centres in London, or those ‘Small Local Centres’, which comprise the largest proportion of retail centres in the UK (Macdonald et al., 2022). However, perhaps the greatest consideration we must make relates to the underlying limitations of the mobility data used in this study, which often has a tendency to introduce generational and/or spatial biases, as identified by Trasberg and Cheshire (2021). However, perhaps the most pressing consideration relates to the temporal stability of the dataset, which as a result of significant reductions in the number of devices and applications over time (Fig. 1), significantly constrained our ability to explore individual trajectories of recovery over time, instead resulting in comparisons between similar areas and modelling of change in the share of activity between two time periods. As a result, there remains significant uncertainty as to the exact nature of retail centre recovery, a major challenge when trying to utilise ‘Big Data’ in Urban Informatics (Shi et al., 2022).

However, given we have devoted significant effort to controlling for the temporal instability of the dataset, through identification of relative stability between all retail centre *functions* and most regions (see Section 4.1), in our opinion the findings we have presented are empirically robust. Whilst there are some important considerations to make about the temporal stability of such data before using it to answer new research questions, correct use of mobility data offers significant advantages over other economic performance measures for retail centres. For example, mobility data does not privilege certain geographic areas or locations within retail centres, as is the case with footfall sensors (Philp et al., 2022), and typically offers a greater temporal resolution than other ‘static’ measures of economic performance, such as vacancy rates (Dolega & Lord, 2020). However, it would still be more preferable to use actual sales data to monetise the performance of retail centres, as is the case with individual stores (e.g., Rose & Dolega, 2022), but the potential to do so has not yet been realised, given a lack of suitable data.

To conclude then, the results of this study provide empirical evidence of the recent recovery of retail centres, highlighting that there are certain *functional,* regional and *structural* characteristics associated with particularly stronger recovery trajectories. Thus, we contribute further to the narrative that retail centre performance is inherently multidimensional (Parker et al., 2017), by showing that various factors including catchment deprivation, centre composition, *function* and regional geography all have a significant role in determining the recovery of retail centres, thus fulfilling the second and third aim of this investigation. In this sense, we argue that national policies seeking to maintain or improve the vitality or viability of consumption spaces need to account for this added knowledge. By taking into account the *functional* role of the retail centre and its *structural* and catchment characteristics, and constructing a ‘Digital Twin’ framework, researchers can use advancements in Big Data and modelling to simulate how such policies can result in positive outcomes for consumption spaces (Goodchild, 2022; Shi et al., 2022). Such interventions have never been more important, as whilst the COVID-19 pandemic remains present, the retail sector is also subject to the recent cost-of-living crisis, where its impacts are already apparent in falling sales and footfall in recent months (ONS, 2022; Wright, 2022). Given the rising costs of energy and food, increasing taxes and wages falling in line with increasing inflation in the UK (Patrick & Pybus, 2022), the retail sector is expected to continue to face some of the most significant impacts, with falls in consumer confidence and a new wave of retail vacancies expected in the near future. This raises significant questions, which are not new, but remain important about the trajectories of retail centre performance in the near future, and the social and economic value that these urban phenomena represent. These issues are however not well understood, and there is a broader agenda for further research into the continued monitoring of retail recovery and decline, utilising retail centre geographies as geographic data tools to provide evidence that can inform policy decisions and provide solutions to both acute and longer-term issues. This study provides an initial basis upon which to do so, through examination of national-level trends in retail centre activity, utilising unstable data derived from mobile phone applications.
